# The first National Family Medicine Conference in Botswana, May 2013

**DOI:** 10.4102/phcfm.v6i1.595

**Published:** 2014-02-21

**Authors:** Vincent Setlhare, Bob Mash, Billy Tsima

**Affiliations:** 1 Department of Family Medicine, University of Botswana, Botswana; 2 Department of Family Medicine and Primary Care, Stellenbosch University, South Africa

## Introduction

The theme of the conference was ‘Family Medicine Training and Career Paths for Family Physicians in Botswana’. This topic was deemed to be appropriate as there is a need for countries to define the role of family physicians and the training requirements for family medicine (FM) in their own contexts.^1,2,3,4^

In 1995, a study showed that there was a need for a medical school in Botswana and planning for this started in 1998.^5^ The plans for a new medical school were driven by the high doctor– patient ratios, the small number of local doctors and the fact that the majority of Botswana doctors graduating overseas did not return to practise. The University of Botswana, School of Medicine, started residency programmes in Internal Medicine and Paediatrics in 2010. Family Medicine and other specialties were started in 2011. The residency programme in Family Medicine had its fair share of teething problems, as was also the case in similar programmes in Kenya and Uganda.^1,2^

The department’s problems stemmed mainly from understaffing and being based in three campuses 200 to 800 kilometres apart. Qualified family physicians were not applying to fill the vacant positions and the two main FM campuses did not have enough specialists in the various disciplines to support the FM residency programme. The requirement of enough specialists to support FM residency programmes is highlighted in the African Consensus Statement on Family Medicine.^3^

The Joint Staff Agreement between the University of Botswana and the Botswana government was supposed to enable non-faculty specialists to dedicate time to teaching residents and undergraduate medical students, but there were problems with its implementation. Training residents under these conditions was a challenge.

In addition, some residents were in their penultimate year of training and their career paths and posts, after completion, were not well defined. There was therefore a need to reconvene stakeholders to share ideas on the role of FM in Botswana, how best to train FM residents and what their career paths would be in the Botswana setting.

## The conference

The conference was held in Gaborone, Botswana from 15-17th May 2013. Conference speakers included the Honourable Minister of Health, a Ministry of Health representative, leading academics in the field of Family Medicine in South Africa, Family Medicine faculty members from the University of Botswana (UB), leading general practitioners (GPs) in Botswana, the Botswana Medical Education Initiative (BoMEPI) representative, and two residents. Sixty people attended the conference daily. They included government officials, UB faculty members, general practitioners, residents and medical students. This composition was similar to, though more inclusive than, that of a Family Medicine workshop in Ethiopia which had similar objectives.^4^

**FIGURE 1:**
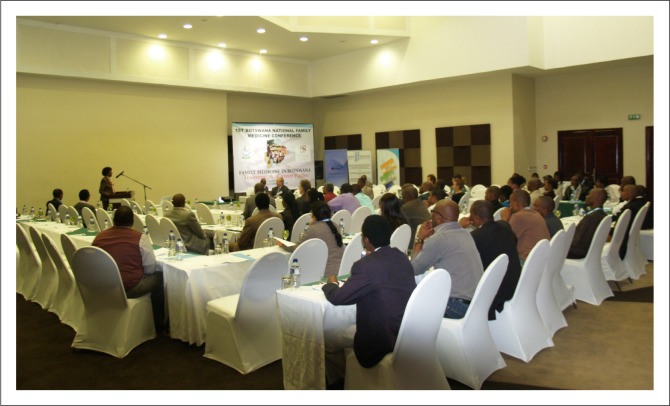
Dr NP Mashalaba, who helped start the Mother and Child Health programmes in Botswana, delivering her presentation.

The conference was divided into seven sessions consisting of plenary sessions and workshops. The first session started with opening remarks by Dr V. Setlhare, the Acting Head of Family Medicine (UB). He highlighted the need for FM to be context embedded and showed that FM is evolving.^2,3^ His remarks were followed by presentations from senior colleagues and leading Botswana GPs (Dr NP Mashalaba [see Figure 1] and Dr K Seligman) who talked about the difficult but enjoyable years of laying the foundations of modern medicine in Botswana. Being overworked in an under-resourced health system and bringing up a young family was a challenge. These founding doctors put together health programmes that have stood the test of time and one talked about how they provided outreach services to remote areas, often sleeping in the bush with the danger of wild animals. They said that their work was inspired by love, not remuneration.

**FIGURE 2:**
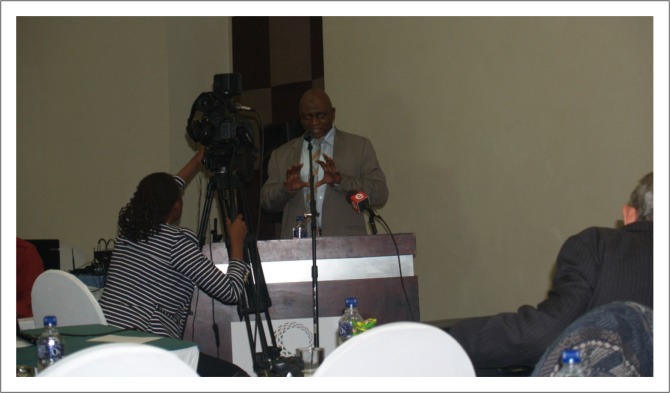
Dr Jibril made a presentation on behalf of the Ministry of Health.

Dr Jibril (see Figure 2), the representative from the Ministry of Health (MOH) showed the structure of the ministry and described its mandate. The strategic themes of the MOH (health promotion, patient care and organisational excellence) were aimed at preventative and curative services as well as patient satisfaction. He saw the role of family physicians as primary care providers, researchers, teachers, policy and strategy formulators, advocates for patients and watchdogs for quality healthcare. He saw family physicians as possessing a comprehensive set of skills that could solve most of the problems of the majority of patients. He endorsed the fact that primary care delivers better health outcomes for communities. Dr Jibril then underlined the fact that family physicians are regarded as specialists by the MOH and are thus remunerated at that rate and progress through the ranks in the same manner as other specialists.

**FIGURE 3:**
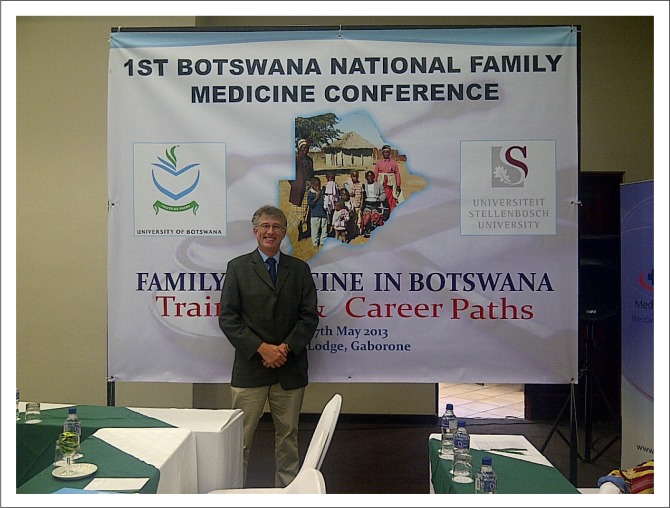
Prof. Bob Mash at the conference.

Prof. Bob Mash (see Figure 3) ended the session with a presentation on the collaboration between Stellenbosch University (SU) and UB Family Medicine departments. The presentation outlined the dearth of doctors in Africa^6^ and acknowledged that FM, or primary care, was best placed to address the health needs of communities.^7^ Family Medicine and primary care were shown to overlap and the presentation defined the scope of FM, the role of the family physician and where he should be trained and work. Prof. Mash posed key questions for the MOH: to define the role of the family physician, where they will work and how many family physician posts are needed. He said that UB needs to satisfy itself that its training outcomes in FM are aligned to the country’s needs and that training is placed correctly within the health system. He also pointed out that UB needed to determine who the FM trainers are, how they are empowered and recognised, the number of residents to be trained in FM to meet the country’s needs and how to ensure that exams assess the correct parameters.

Breakout sessions addressed pertinent issues relating to undergraduate and postgraduate training in FM in Botswana. Participants in both groups brainstormed ideas to inform future training in family medicine and these were reported back to the group as a whole. Recommendations from the group sessions informed parts of the consensus statement that was adopted by participants as ‘the Gaborone consensus’. Time was also allocated for oral presentations from clinicians and FM residents, with topics including clinical medicine and preliminary reports from MMed dissertations.

In the second session, Prof. SS (Cyril) Naidoo started his presentation with a moving inspirational story of an underachieving underprivileged child raised by a single mother. She transformed him into an A student who became one of the leading lights in neurosurgery worldwide. He highlighted the inefficiencies of the South African public health system and how there was a renewed effort to address this by implementing an effective primary healthcare initiative. Prof. Naidoo said that some of the challenges of FM training are inadequate staffing, poor salaries, unattractive career paths and the burden of care and responsibility.

In the third session, Dr Nkomazana related how FM started in Botswana with the involvement of Stellenbosch University. She related how stakeholders assembled in 2008 in a workshop to help start FM training, which workshop was immediately followed by recognition of FM as a specialty by the Botswana Health Professions Council (BHPC) and the start of a process of creating family physician posts in the public sector. Dr Nkomazana showed the important role that BoMEPI had played in equipping FM training sites.

Prof. Ogunbanjo showed that although Botswana was a middle-income country, doing well with regard to childhood immunisation and antenatal care, life expectancy and health expenditure were both low. Healthcare worker to population ratios and contraception uptake were low, whilst its HIV and TB prevalence were amongst the highest in the world. He showed that Botswana has a strong primary healthcare infrastructure and that the main contributors to disability adjusted life years (DALYS) were diarrhoea, respiratory infections and injuries. Prof. Ogunbanjo said South Africa has adopted primary healthcare as the pillar or foundation of its healthcare system. He said that family physicians should be trained to manage themselves and teams with visionary leadership, to provide high quality evidence-based care, to teach and to conduct themselves both ethically and professionally. He recommended that the role for and career paths of family physicians in the public sector needed to be clarified operationally by the end of 2014 when the first cohort of FM residents graduates. Prof. Ogunbanjo recommended that FM training sites be expanded to all districts and that the MOH should help UB in recruiting FM trainers so that the staff–resident ratio could be at least 1 supervisor to 4 residents. He recommended more scholarships for FM residents, yearly FM conferences and the formation of strategic advocacy platforms for FM, such as a College of Family Physicians of Botswana.

Dr Setlhare showed that the rural to urban distribution of the population of Botswana is 1:4 and that almost half of the people of Botswana live within 70 kilometres of Gaborone.^8^ He also showed that some of the most under-served areas in the health system are within this Gaborone area, pointing out that there are relatively few health facilities within the area given that it contains nearly half the population.^9^ Dr Setlhare also showed the potential for FM training in this area, which has five primary hospitals and many clinics within easy reach of Gaborone. This has important implications for family physicians with young families who would like to join the department as trainers, but would not like to be posted far from Gaborone where facilities are inadequate. He noted that the department should set clear training goals that address Botswana’s health needs and that trainers should supervise rather than teach, using problem-based and self-directed learning as the underlying philosophy. The importance of frequent supervised presentations by residents and exposing them to other FM departments through visits was deemed to be of great importance.

Prof. Blitz talked about professionalism and its definition in terms of social accountability. It can also be seen as the aims, conduct and qualities that mark a profession or a professional person. She reported on a study in the United Kingdom that revealed that absenteeism was the most common breach of professionalism in medical school. Such breaches need to be identified and discussed and remedial action taken. Professionalism can be taught in the curriculum, but it is ‘caught’ from observing what takes place in the work environment. Prof. Blitz said professionalism can be improved by spelling out expectations, improving the work environment and by making the hidden curriculum visible.

**FIGURE 4:**
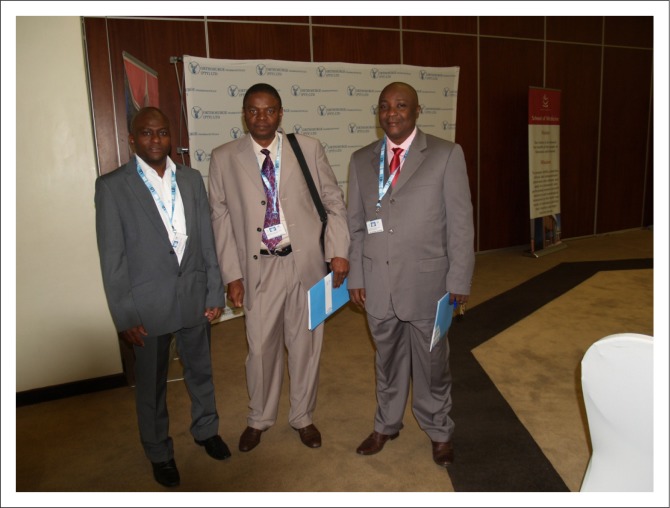
Faculty of the Department of Family Medicine (University of Botswana), Doctors Ganiyu, Mbuka and Tshitenge, at the conference.

Other speakers were Doctors L Parsons, J Mwita, A Ganiyu, D Mbuka, T Tsima, S Tshitenge, J Firth and M Reid (see Figure 4). The conference adopted the consensus document laid out below.

## First Botswana National Family Medicine Conference

### Consensus document

The participants of the first Botswana National Family Medicine Conference (BNFMC) acknowledge the positive achievements of the government of Botswana and the University of Botswana (UB) in:

- Establishing Family Medicine in Botswana.
- Establishing a Department of FM at UB – both undergraduate and post graduate programmes.
- Gaining recognition by BHPC.
- Establishing the MMed programme.
- Obtaining approval for residents to write the CMSA (College of Medicine of South Africa) exit examination in Family Medicine.
- Creation of residency posts by government.

In order to develop FM further, the following conditions need to be met by the University of Botswana and the Ministry of Health in Botswana:

- A new training complex needs to be developed in or around Gaborone and existing training complexes need to be retained.
- Adequate resources need to be made available in order to develop and retain these training complexes, including adequate specialist staff at district hospitals.
- FM specialist posts in primary healthcare and primary hospitals need to be created by the end of 2014 by the MOH.
- The Joint Service Agreement needs to be revived and applied so that the number of health workers involved in training residents in the workplace is increased.
- The MOH needs to create a policy document on the expected role of a family physician in the Botswana health system.
- The MOH needs to create conditions conducive to the recruitment and retention of residents.
- The location of FM training should aim to shift to primary hospitals and primary healthcare (PHC) settings.
- There should be an annual FM conference in Botswana.
- A professional body for FM should be established in Botswana.
- The internship programme needs to include an FM/PHC component.
- The involvement of private practitioners in the training programmes should be considered.
